# Deficiency of MIWI2 (Piwil4) Induces Mouse Erythroleukemia Cell Differentiation, but Has No Effect on Hematopoiesis *In Vivo*

**DOI:** 10.1371/journal.pone.0082573

**Published:** 2013-12-23

**Authors:** James E. Jacobs, Mark Wagner, Joseph Dhahbi, Dario Boffelli, David I. K. Martin

**Affiliations:** Children's Hospital Oakland Research Institute, Oakland, California, United States of America; Victor Chang Cardiac Research Institute, Australia

## Abstract

Piwi proteins and their small non-coding RNA partners are involved in the maintenance of stem cell character and genome integrity in the male germ cells of mammals. MIWI2, one of the mouse Piwi-like proteins, is expressed in the prepachytene phase of spermatogenesis during the period of de novo methylation. Absence of this protein leads to meiotic defects and a progressive loss of germ cells. There is an accumulation of evidence that Piwi proteins may be active in hematopoietic tissues. Thus, MIWI2 may have a role in hematopoietic stem and/or progenitor cell self-renewal and differentiation, and defects in MIWI2 may lead to abnormal hematopoiesis. MIWI2 mRNA can be detected in a mouse erythroblast cell line by RNA-seq, and shRNA-mediated knockdown of this mRNA causes the cells to take on characteristics of differentiated erythroid precursors. However, there are no detectable hematopoietic abnormalities in a MIWI2-deficient mouse model. While subtle, non-statistically significant changes were noted in the hematopoietic function of mice without a functional MIWI2 gene when compared to wild type mice, our results show that MIWI2 is not solely necessary for hematopoiesis within the normal life span of a mouse.

## Introduction

Small, single-stranded RNA molecules of approximately 20–30 nucleotides (nt) have been discovered in a wide spectrum of species [1]–[5]. In association with specific proteins, small non-coding RNAs (ncRNAs) have been shown to be involved in transcriptional regulation, chromatin structural organization and mRNA stability. Several classes of small ncRNAs act as sequence guides that direct members of the Argonaute protein family, and their associated protein complexes, to partially or fully complementary nucleic acids. The Argonaute family is divided into two major clades: Ago proteins and Piwi proteins [6],[7]. Micro RNAs (miRNAs) and small inhibiting (siRNAs) associate with Ago proteins to target mRNAs or viral genomes [1],[2],[7]. Piwi and its associated RNAs (piRNAs) have less well-defined functions [1],[2]. They are linked to the maintenance of stem cell character and genome integrity [5], but the mechanisms by which they mediate these effects are not completely understood.

Expression of Piwi proteins and piRNAs was thought to be largely restricted to germ cells and further restricted in mammals to male germ cells [1],[3],[8]. However, several lines of evidence have suggested that they may be active in other cellular systems as well. Traces of Piwi-like protein expression have been detected in human CD34^+^ hematopoietic progenitor cells [9]. A 28-bp piRNA-like small RNA is involved in the CpG methylation of one of the killer Ig-like receptor (KIR) gene promoters in natural killer (NK) cells [10]. Furthermore, expression of small RNAs with piRNA-like features has been noted in various somatic tissues [11],[12].

The biological roles of piRNAs are likely diverse, as suggested by the analysis of genomic mapping of annotated piRNAs. In mice, piRNAs can be subdivided into pachytene and prepachytene based on the timing of their expression during spermatogenesis. Expression of prepachytene piRNAs can be detected at embryonic day 16.5 (E16.5). Levels peak around the time of birth, and then decrease; possibly forming a minor subpopulation in mature testis [2],[13]. Prepachytene piRNAs are largely derived from retrotransposon sequences, and are believed to participate in silencing of active retrotransposons either by cleavage of their transcripts or by direct recruitment of epigenetic modifications [1]–[3],[8],[14]. In contrast, pachytene piRNAs are derived from genomic regions comprised of unique sequences that are devoid of retrotransposons. In the mouse, their expression begins around 14 days post partum (dpp) and corresponds with the third phase of meiotic prophase I. These piRNAs lack obvious complementary sites in the genome, and their functions are obscure [1],[2],[4],[14],[15].

The ability of stem cells to self-renew is important in a variety of biological systems including germ cells, and cells of the hematopoietic system. Argonaute proteins have a role in stem cell maintenance in many widely divergent species [3],[6],[16], and this has been most intensively investigated in the germ cells of *Drosophila*, zebrafish and male mice. In *Drosophila*, PIWI is required for the self-renewal of germ-line stem cells, and flies deficient in PIWI show progressive differentiation of the germ cells without maintenance of the stem cell pool [16]. Interestingly, the functions of PIWI are not dependent on its endonuclease activity, suggesting that its functions are mediated by its participation in a larger regulatory complex [17]. In the mouse, the Piwi clade has three known functional members: MIWI (PIWIL1), MILI (PIWIL2), and MIWI2 (PIWIL4). MIWI is expressed in the male gonads during the pachytene stage of spermatogenesis, beginning 2 weeks postnatally [13],[18],[19]. Male mice deficient in MIWI show arrest of spermatogenesis at the beginning of the round spermatid stage [6]. MILI is expressed in both male and female primordial germ cells (PGCs) at E12.5, and its expression declines as germ cells develop into mature gametes [8],[13],[18]. MIWI2 is expressed briefly in the prepachytene stage of spermatogenesis, beginning at E15.5 during the critical period of de novo methylation [7],[8],[13]. Male mice deficient in functional MIWI2 demonstrate meiotic defects in prophase of meiosis I, followed by a progressive loss of germ cells over time. These defects have been discovered in association with activation of transposable elements that are normally silent in developing germ cells [7]. Further study suggests that in the case of MILI and MIWI2, the defects in spermatogenesis are secondary to decreased de novo methylation of transposable elements in the germline [8],[13].

Given the role of Piwi proteins in the process of germ cell development, it is possible that other stem cell populations have similar regulatory mechanisms in place. Several biological systems rely on the ability of pluripotent stem cells to self-renew and differentiate. The hematopoietic cells – one of the most prolific cellular systems in mammals – are a prominent example [20]. Data from the ENCODE project indicates expression of MIWI2 in mouse spleen and in mouse erythroleukemia (MEL) cells, with minimal expression of MIWI and MILI [21],[22]. The expression of MIWI2 in blood cells and blood-forming tissues suggests a possible role for Piwi proteins in hematopoiesis. To investigate this, we used a cell culture model in which MIWI2 expression is knocked down by shRNA, and a mouse model in which the animals lack a functional MIWI2 gene to test the hypothesis that MIWI2 functions in hematopoietic stem and/or progenitor cell self-renewal and differentiation, and that inactivation of MIWI2 results in abnormal hematopoiesis. The results of these studies suggest an effect of MIWI2 deficiency on erythroid differentiation *in vitro*. However, an *in vivo* effect was not observed. While our findings add to the mounting evidence for non-germ-cell functions of Piwi proteins in mammals, they do not indicate that MIWI2 is required for normal hematopoiesis.

## Results

### Tissue-specific expression of MIWI2 mRNA by RNA-seq

**Figure 1:**
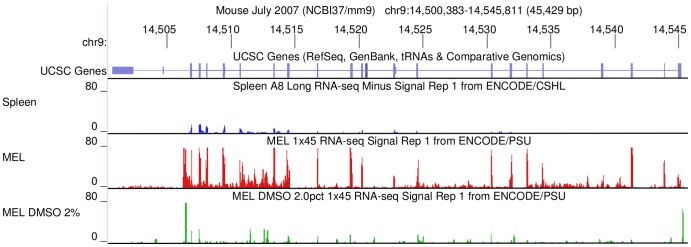
Expression of MIWI2 in spleen and MEL cells. The top track shows the MIWI2 (PIWIL4) gene, as depicted by the UCSC Genes track on the UCSC Genome Browser [21]. The gene itself is depicted in purple and its position on chromosome 9 in shown. Note the 3′ untranslated region at the far left. Below are ENCODE [22] data tracks representing RNA-seq signals from adult spleen (blue), uninduced MEL cells (red) and MEL cells induced with 2% DMSO (green). Uninduced MEL cells show a relatively strong signal for MIWI2 when compared to the low – but present – signal in induced MEL cells and spleen.

We searched for evidence of MIWI2 transcription in hematopoietic tissue by utilizing the publicly available RNA-seq datasets from the ENCODE database on the UCSC Genome Browser [21],[22]. The density of RNA-seq mapped reads (signal) for spleen and MEL cells were compared to the UCSC gene annotation of MIWI2 (Figure 1). Uninduced MEL cells show a relatively strong signal for MIWI2 mRNA transcription. This signal is decreased upon induction of differentiation with dimethyl sulfoxide (DMSO). Spleens from 8 week old mice show a weak but detectable signal as well. Expression of MILI and MIWI by RNA-seq is either extremely low or absent in MEL cells and adult spleen (not shown).

MEL cells are Friend virus-transformed mouse erythroblasts whose differentiation is blocked at a very early (CFU-E) stage. MEL cells will proliferate indefinitely in their undifferentiated state, but undergo erythroid differentiation when treated with certain chemical agents such as DMSO [23],[24]. Cells at the CFU-E stage of erythropoiesis are exceedingly rare in the adult mouse spleen, which is mostly populated with erythroid lineage precursors at later stages of development. Upon treatment with DMSO, MEL cells take on more differentiated characteristics which resemble the red blood cell precursors of the adult spleen. Thus, it is not surprising that induced MEL cells and adult spleen cells show a similar pattern of MIWI2 mRNA expression.

### shRNA-mediated knockdown of MIWI2 in MEL cells

**Figure 2:**
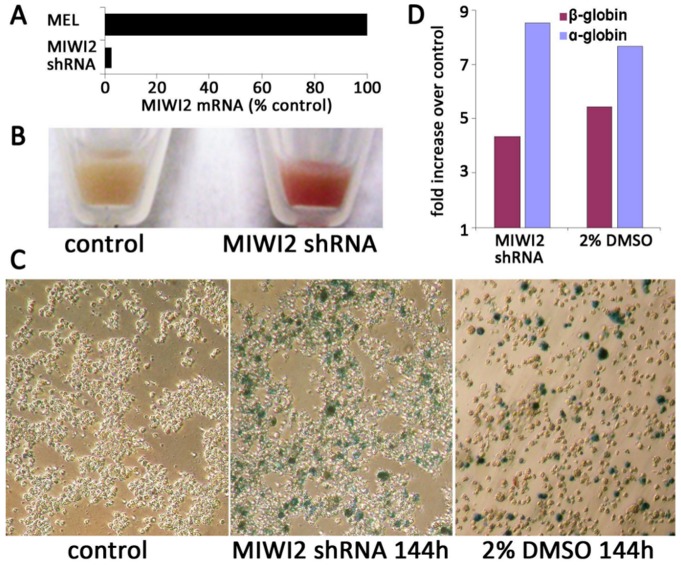
Knockdown of MIWI2 mRNA by RNAi induces differentiation of MEL cells. [A] 48 h after infection of MEL cells with a lentiviral vector expressing a shRNA specific for MIWI2, the level of MIWI2 mRNA is sharply lower. [B] Pelleted control MEL cells (left) and MEL cells 144 h post infection with the MIWI2-specific shRNA lentiviral vector (right). The distinctly red color of the cells at right indicates erythroid differentiation with hemoglobin expression. [C] Benzidine stained control MEL cells (left), MIWI2 shRNA knockdown cells 144 h after infection (center), and MEL cells induced with DMSO for 144 h (right). Following staining with benzidine cells that express hemoglobin appear blue. [D] Expression of α-globin (Hgα) and ßmaj-globin (Hgβ) mRNA in MEL cells 144 h after beginning knockdown of MIWI2 (top), or 144 h after beginning induction of terminal differentiation with 2% DMSO (bottom). mRNA expression was measured by quantitative RT-PCR; levels are expressed relative to untreated MEL cells, normalized to GAPDH control.

Given the finding that RNA-seq identifies MIWI2 mRNA expression in MEL cells, an *in vitro* model was designed to directly probe the role of MIWI2 in MEL cell differentiation. We used shRNA-expressing lentiviral constructs that specifically target the MIWI2 mRNA for degradation by RNAi (Figure 2A). Knockdown of MIWI2 drives differentiation of MEL cells with an efficiency similar to that of DMSO (Figure 2B–D). The cells become red, and show an increase in the expression of hemoglobin as assayed by benzidine staining. They also demonstrate a several-fold increase in expression of α- and ß-globin by qRT-PCR. Lentiviral constructs expressing shRNAs targeting human MLH1, as well as a non-silencing lentiviral construct using a scrambled shRNA have no effect on MEL cells (not shown). Furthermore, other MIWI2-specific shRNA constructs also induce differentiation (not shown), indicating that the differentiation is a specific effect of MIWI2 knockdown.

### MIWI2-deficient Mouse Model

To study the effects of MIWI2 deficiency on hematopoiesis *in vivo*, we made use of a previously developed knockout model in which a targeting vector is inserted between exons 1 and 3 of the MIWI2 gene, causing the deletion of exon 2 [8]. The mutant allele is maintained on a C57BL/6J background. Mice lacking a functional MIWI2 gene are designated as MIWI2*^−/−^* and wild type controls as MIWI2^+/+^. The reports describing this knockout model, and another similar model [7], did not note effects on any tissue other than male germ cells. However, a subtle defect in hematopoiesis might easily go unnoticed, particularly if it did not cause overt anemia or immunodeficiency. For this reason we designed studies that could detect slight defects in hematopoietic lineages.

### Effects of MIWI2 on Hemoglobin Composition in Mice

Given the finding that knockdown of MIWI2 increased hemoglobin production in MEL cells (Figure 2), we evaluated the effects of MIWI2 on the ability of mice to achieve a normal adult hemoglobin profile. We compared hemoglobin types in MIWI2*^−/−^* mice with those of MIWI2^+/+^ mice using high-performance liquid chromatography. The various hemoglobin molecules were identified based on their relative retention times and quantified based on the area under the curve. No differences in hemoglobin patterns were noted between the knockout and wild type mice (Figure S1 in File S1).

### Effect of Age on Hematopoiesis in MIWI2-Deficient Mice

**Figure 3:**
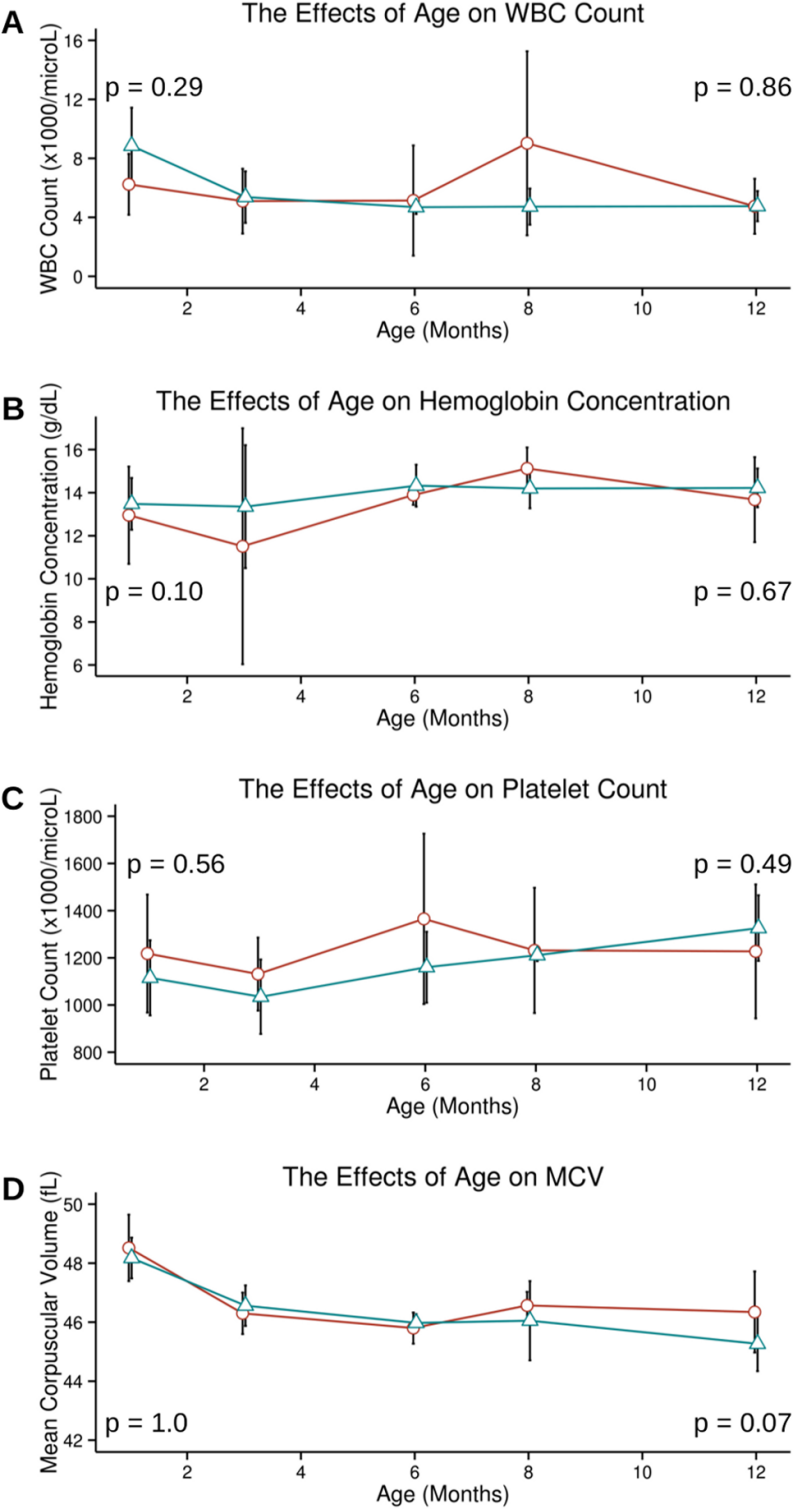
The effects of age on blood cell indices. C57BL/6J mice were mated with mice harboring a heterozygous knockout of MIWI2. Blood indices from homozygous knockout progeny (red circles) are compared with wild type progeny (blue triangles) at 1, 3, 6, 8 and 12 months of life. The blood cell indices include [A] white blood cell count, [B] hemoglobin concentration, [C] platelet count, and [D] mean corpuscular volume. P-values are given for the 1 month and 12 month time points.

As noted previously, loss of MIWI2 in male mice results in failure of germ cells to differentiate properly, and additionally leads to the gradual loss of germ cells over time [7]. Blood cell development is a lifelong process, requiring constant replenishment of lineage progenitors from the pool of HSCs. If MIWI2 has a role in HSC maintenance similar to that in male germ cells, a hematopoietic defect in MIWI2*^−/−^* mice might not be evident until many cycles of differentiation and self-renewal have taken place, causing the gradual appearance of measurable defects in hematopoiesis as mice age. In order to investigate the longitudinal effects of MIWI2 deficiency on the hematopoietic system, the hematologic indices of MIWI2*^−/−^* mice were evaluated at 1 month, 3 months, 6 months, 8 months and 1 year of age, and compared with MIWI2^+/+^ mice of the same age. The blood indices were compared using a two-sample Wilcoxon rank sum test (2-sided). No significant differences in WBC count, hemoglobin concentration, mean corpuscular volume or platelet count were detected over time (Table S1 in File S1 and Figure 3). However, MIWI2*^−/−^* mice showed a trend towards a higher MCV at the 12-month time point that nearly reached statistical significance (p = 0.07).

### Effects of MIWI2 Deficiency on Hematopoietic Recovery in Mice Following Sublethal Irradiation

**Figure 4:**
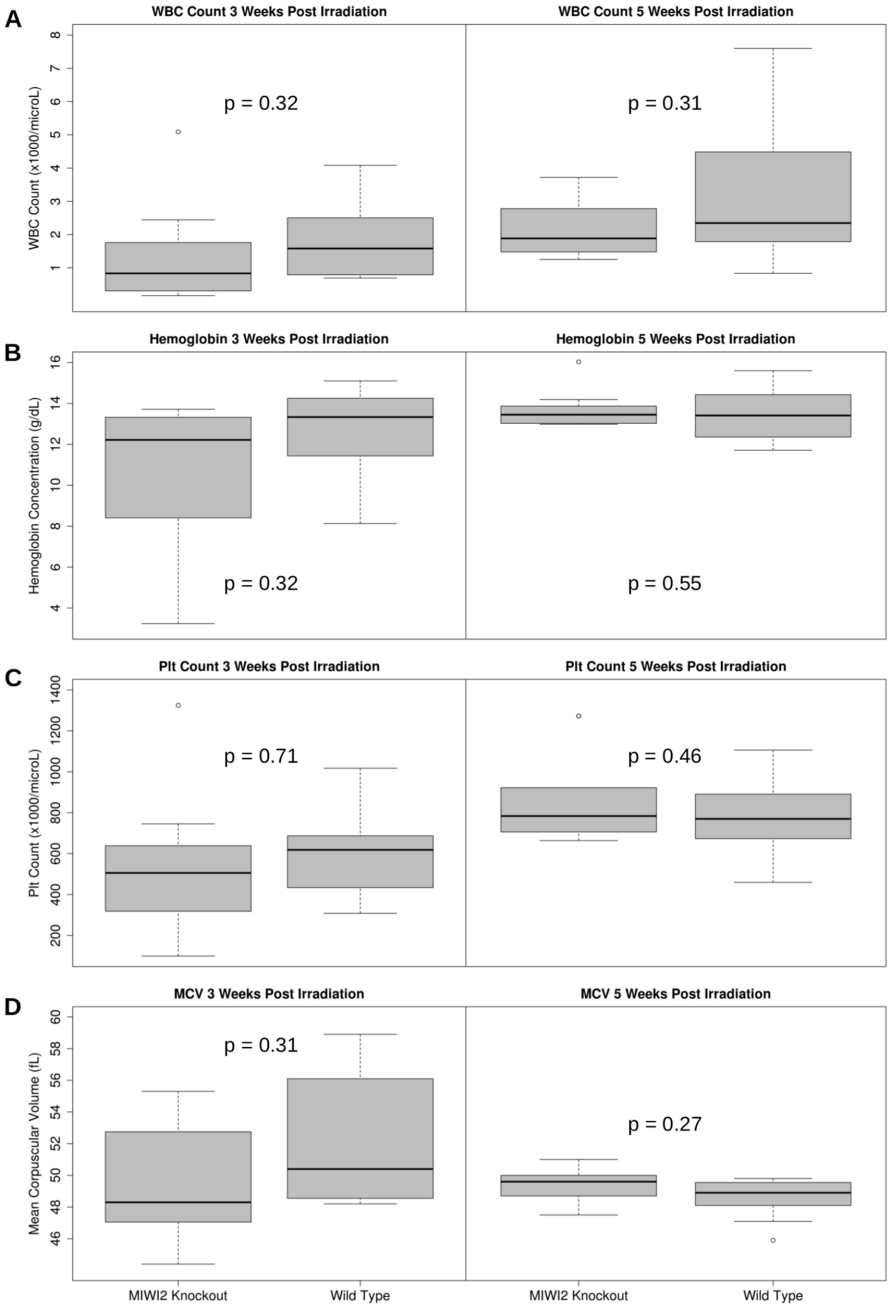
The effects of sublethal irradiation on blood cell indices. C57BL/6J mice were mated with mice harboring a heterozygous knockout of MIWI2. Mice were subjected to a sublethal dose of irradiation (500 cGy). Blood indices from homozygous knockout progeny (labeled “MIWI2 Knockout”) are compared with wild type progeny at 3 weeks and 5 weeks post irradiation. The blood cell indices include [A] white blood cell count, [B] hemoglobin concentration, [C] platelet count, and [D] mean corpuscular volume. P-values indicate the comparison between wild type and knockout mice at the given time point.

As the system of continued blood cell development throughout the life of an organism is a complex process, it is possible that MIWI2 may act in a redundant fashion alongside other regulatory elements. By exposing the hematopoietic system to stress in the form of sublethal irradiation, subtle effects on hematopoietic regeneration can be measured. To study the hematopoietic regenerative potential of mice lacking MIWI2, X-irradiation was used to stress the mouse hematopoietic system. MIWI2^+/+^ and MIWI2*^−/−^* mice (8–12 weeks of age) were irradiated with 500cGy, a dose sufficient to destroy the majority of hematopoietic progenitors, but leave enough HSCs to allow spontaneous complete recovery of hematopoiesis [25]. The hematologic indices of the mice were evaluated at 3 weeks and 5 weeks after irradiation. At 3 weeks post-irradiation, there was an overall trend towards a lower hemoglobin concentration, lower MCV, lower platelet count, and lower WBC count in the MIWI2*^−/−^* mice (Figure 4), but the differences did not reach statistical significance. With the exception of the WBC count, these trends were largely absent at 5 weeks post-irradiation. The MIWI2*^−/−^* mice continued to show a trend towards a lower WBC count at the 5 week post-irradiation time point, again not attaining statistical significance.

## Discussion

Hematopoiesis is a complex and dynamic process in which multipotent HSCs are required to self-renew and – under the proper stimuli – differentiate into a diverse population of progenitor cells that ultimately give rise to the heterogeneous mature peripheral blood cells [20],[26]. Further complexity is added because hematopoiesis is not a static system. Shifting demands for various blood cell types must constantly be met, and regulatory fine-tuning of this intricate system requires both cell autonomous and non-autonomous factors from the HSC niche. Given the parallels between male germ cells and HSCs with respect to self-renewal and differentiation, and based on evidence that the piRNA pathway is active in somatic cells, we sought to identify abnormalities of blood cells deficient in MIWI2.

MIWI2 mRNA is transcribed in MEL cells, and knockdown of MIWI2 in this hematopoietic cell line causes the same characteristic changes seen in DMSO-induced differentiation, indicating that MIWI2 has a functional role in hematopoiesis *in vitro*. However, subsequent *in vivo* experiments failed to detect any statistically significant differences in hematopoiesis when MIWI2 is non-functional. When the hematopoietic system of mice is stressed with sublethal irradiation, MIWI2*^−/−^* mice show a non-significant trend toward slower recovery of all four blood indices at the 3 week post-irradiation time point when compared to irradiated MIWI2^+/+^ mice. These results indicate that either the absence of MIWI2 does not lead to a decreased ability to recover following hematopoietic stress, or that the effect of MIWI2 deficiency is mild. Our experiments also demonstrate that aged MIWI2*^−/−^* mice show a trend (also non-significant) towards a higher MCV when compared with MIWI2^+/+^ controls (p = 0.07 at 12 months of life). Elevated MCV can be a sign of stress erythropoiesis and is seen as a downstream phenotype from various causes such as nutritional deficiencies (folate and Vitamin B12) or aplastic anemia. The lack of *in vivo* effects of MIWI2 deficiency on the hematopoietic system suggest that while there may be a role for MIWI2 in hematopoietic regulation – as suggested by the effects of knockdown on MEL cells – this role is not critical to normal hematopoiesis or to recovery following hematopoietic stress. During the review of this paper, Nolde and colleagues published their findings regarding the function of Piwi proteins in mouse hematopoiesis. They established that hematopoietic function of mice was intact, even in the setting of a triple knockout (MIWI, MIWI2 & MILI) [27]. Nolde's results support the interpretation of our data that Piwi proteins are not essential to mouse hematopoiesis *in vivo*. However, our finding that knockdown of MIWI2 in MEL cells induces the cells to take on characteristics of differentiated erythroid cells (Figure 2) brings to light the possibility that Piwi proteins may have a role in erythroid differentiation.

Our results do not confirm a difference between the hematopoietic systems of MIWI2*^−/−^* and MIWI2^+/+^ mice, although we note a global trend in all hematopoietic cell lines towards decreased recovery at 3 weeks post-irradiation. A MEL cell model produced a dramatic effect on differentiation, but the complexity of hematopoiesis demands the use of an *in vivo* model and in this model no statistically significant effects were apparent. Our analysis was limited by the use of only two post-irradiation time points and relatively small numbers (n: 4–9 in aging analysis at 1 and 12 months; n: 7–12 in sublethal irradiation analysis). As hematopoietic recovery was well underway at 3 weeks post-irradiation, a more substantial effect might have been appreciable earlier in the recovery period. We conclude that an effect of MIWI2 knockout on hematopoiesis – if there is any – is too subtle to detect without the use of larger numbers of animals.

MIWI2 is expressed in differentiating hematopoietic cells. The evidence that it is required for maintenance of undifferentiated MEL cells, but not for normal hematopoiesis *in vivo*, leaves open the question of what function it may have. While it is tempting to speculate that a somatic function of MIWI2 may be redundant with other Piwi proteins, expression of MIWI and MILI are absent or extremely low in spleen and MEL cells. However, earlier studies indicated that HIWI is highly expressed in human hematopoietic stem cells, suggesting that it may function at earlier stages of hematopoiesis. Further investigation will thus be required to clarify the role of the Piwi-piRNA pathway in hematopoiesis.

## Materials and Methods

### Ethics Statement

All animal procedures were performed in strict accordance with the policies set forth in the Children's Hospital Oakland Research Institute Animal Care and Use Handbook. The protocol was approved by the Institutional Animal Care and Use Committee of Children's Hospital Oakland Research Institute (Animal Welfare Assurance Number: A3631-01). Ear tag placement, tail clipping and blood draws were performed under isoflurane anesthesia, and all efforts were made to minimize suffering.

### Mice

Mice with a heterozygous deficiency of MIWI2 (MIWI2*^+/−^*) [8] were kindly provided by Haig Kazazian, and bred with C57BL/6J mice in standard conditions to obtain homozygotes (MIWI2^−*/−*^).

### DNA Isolation and Genotyping

Samples for genotyping were obtained via tail clipping. DNA was purified using QIAGEN DNeasy Blood and Tissue Kit. The purified DNA was PCR amplified. The amplified product was then run on a 1% agarose gel after being stained with ethidium bromide. The wild type MIWI2 PCR product produces a band at 540 bp. The knockout PCR product produces a band at 300 bp. Thus, by comparing the bands to a standard DNA ladder MIWI2^+/+^, MIWI2^+/−^ and MIWI2^−/−^ can be identified. The primers are as follows: Miwi2-8AS (5′-GTCCACCATCACCAGAAG-3′), pPNT-1 (5′-CCTACCCGGTAGAATTGACC-3′), Miwi2-int2 (5′-AGTACCTTCCAAGTGGTG-3′).

### Blood Analysis

Blood samples (average volume of 75–100 µL) were obtained from the submandibular venous plexus under general anesthesia. The blood was processed in a PBS-based buffer (330 mOsmol and pH 7.4) with EDTA as an anticoagulant. These samples were run on the ADVIA 120 Hematology System (Siemens), providing complete blood counts including counts of white blood cells, red blood cells, and platelets; measurement of the hemoglobin concentration as well as the mean corpuscular volumes.

### Irradiation

Sublethal irradiation (500cGy of total body ionizing irradiation) was administered (RADSource 2000, X-ray biological irradiator, 160 kV, 4.2 kW, 25 mA) to 8–12 week old mice. The hematologic parameters of mice were then evaluated at 3 and 5 weeks post irradiation.

### Informatics Analysis

The MIWI2 gene was viewed on the Mouse July 2007 (NCBI37/mm9) Assembly in the UCSC Genome Browser [21]. Expression and Regulation tracks include 8 week old C57BL/6J mouse spleen cells from the Cold Spring Harbor Laboratories Long RNA-seq dataset and MEL cells (uninduced and induced) from the PSU RNA-seq dataset. The cell lines were prepared and processed as described on the UCSC Genome Browser website at http://genome.ucsc.edu.

### shRNA-mediated knockdown

293T packaging cells were co-transfected with lentiviral shRNA vector targeting MIWI2 (Open Biosystems), and with pMD2 and psPAX2 packaging vectors (Addgene). Medium was harvested and supplemented with polybrene for lentiviral infection of MEL cells, followed by selection in medium containing puromycin (1 μg/ml) starting at 48 h as described [28]. Results presented are from three or more independent experiments in which all studies described were performed in triplicate; representative images are shown. Infection with lentiviral vectors expressing shRNAs specific to another mRNA (hMLH1) had no effect on MEL cells. Similarly, a lentiviral vector expressing a scrambled shRNA (non-silencing) had no effect on MEL cells. The MIWI2-specific shRNA sequences can be found at http://www.thermoscientificbio.com/shrna/trc-lentiviral-shrna/?term=PIWIL4&productId=1961A1D5-D4C1-4A23-A7D0-81D8B30525CB&sourceId=EG/143689.

### Benzidine staining

Cells were suspended in 100 μl of PBS and 100 μl of benzidine. Working solution containing 4 μl H_2_O_2_ was added to the suspension, which was incubated at room temperature for 2 min. Cells were counted using a hemacytometer, or photographed on a glass slide.

### Statistical Analysis

Statistical analysis was performed using the R software package (version 2.14.1 for linux; Copyright 2011, The R Foundation for Statistical Computing, Vienna, Austria). All p-values were calculated using a two-sided Wilcoxon rank sum test.

## Supporting Information

File S1 **Supporting Documents.** Figure S1. High-Performance Liquid Chromatography of Peripheral Blood from Wild Type and Homozygous MIWI2 Knockout Mice. Table S1. The Effects of Age on Blood Cell Indices.
(DOCX)

## References

[1] Aravin AA, Hannon GJ, Brennecke J (2007) The Piwi-piRNA pathway provides an adaptive defense in the transposon arms race. Science 318: 761–764. 17975059 10.1126/science.1146484

[2] Ghildiyal M, Zamore PD (2009) Small silencing RNAs: an expanding universe. Nat Rev Genet 10: 94–108. 19148191 10.1038/nrg2504 PMC2724769

[3] Houwing S, Kamminga LM, Berezikov E, Cronembold D, Girard A, et al (2007) A role for Piwi and piRNAs in germ cell maintenance and transposon silencing in Zebrafish. Cell 129: 69–82. 17418787 10.1016/j.cell.2007.03.026

[4] Robine N, Lau NC, Balla S, Jin Z, Okamura K, et al (2009) A broadly conserved pathway generates 3′UTR-directed primary piRNAs. Curr Biol 19: 2066–2076. 20022248 10.1016/j.cub.2009.11.064 PMC2812478

[5] Zamore PD, Haley B (2005) Ribo-gnome: the big world of small RNAs. Science 309: 1519–1524. 16141061 10.1126/science.1111444

[6] Carmell MA, Xuan Z, Zhang MQ, Hannon GJ (2002) The Argonaute family: tentacles that reach into RNAi, developmental control, stem cell maintenance, and tumorigenesis. Genes Dev 16: 2733–2742. 12414724 10.1101/gad.1026102

[7] Carmell MA, Girard A, van de Kant HJ, Bourc'his D, Bestor TH, et al (2007) MIWI2 is essential for spermatogenesis and repression of transposons in the mouse male germline. Dev Cell 12: 503–514. 17395546 10.1016/j.devcel.2007.03.001

[8] Kuramochi-Miyagawa S, Watanabe T, Gotoh K, Totoki Y, Toyoda A, et al (2008) DNA methylation of retrotransposon genes is regulated by Piwi family members MILI and MIWI2 in murine fetal testes. Genes Dev 22: 908–917. 18381894 10.1101/gad.1640708 PMC2279202

[9] Sharma AK, Nelson MC, Brandt JE, Wessman M, Mahmud N, et al (2001) Human CD34(+) stem cells express the hiwi gene, a human homologue of the Drosophila gene piwi. Blood 97: 426–434. 11154219 10.1182/blood.v97.2.426

[10] Cichocki F, Lenvik T, Sharma N, Yun G, Anderson SK, et al (2010) Cutting edge: KIR antisense transcripts are processed into a 28-base PIWI-like RNA in human NK cells. J Immunol 185: 2009–2012. 20631304 10.4049/jimmunol.1000855 PMC3477858

[11] Yan Z, Hu HY, Jiang X, Maierhofer V, Neb E, et al (2011) Widespread expression of piRNA-like molecules in somatic tissues. Nucleic Acids Res 39: 6596–6607. 21546553 10.1093/nar/gkr298 PMC3159465

[12] Ro S, Park C, Song R, Nguyen D, Jin J, et al (2007) Cloning and expression profiling of testis-expressed piRNA-like RNAs. RNA 13: 1693–1702. 17698640 10.1261/rna.640307 PMC1986815

[13] Aravin AA, Sachidanandam R, Bourc'his D, Schaefer C, Pezic D, et al (2008) A piRNA pathway primed by individual transposons is linked to de novo DNA methylation in mice. Mol Cell 31: 785–799. 18922463 10.1016/j.molcel.2008.09.003 PMC2730041

[14] Aravin AA, Sachidanandam R, Girard A, Fejes-Toth K, Hannon GJ (2007) Developmentally regulated piRNA clusters implicate MILI in transposon control. Science 316: 744–747. 17446352 10.1126/science.1142612

[15] Aravin A, Gaidatzis D, Pfeffer S, Lagos-Quintana M, Landgraf P, et al (2006) A novel class of small RNAs bind to MILI protein in mouse testes. Nature 442: 203–207. 16751777 10.1038/nature04916

[16] Cox DN, Chao A, Baker J, Chang L, Qiao D, et al (1998) A novel class of evolutionarily conserved genes defined by piwi are essential for stem cell self-renewal. Genes Dev 12: 3715–3727. 9851978 10.1101/gad.12.23.3715 PMC317255

[17] Darricarrere N, Liu N, Watanabe T, Lin H (2013) Function of Piwi, a nuclear Piwi/Argonaute protein, is independent of its slicer activity. Proc Natl Acad Sci U S A 110: 1297–1302. 23297219 10.1073/pnas.1213283110 PMC3557079

[18] Kuramochi-Miyagawa S, Kimura T, Yomogida K, Kuroiwa A, Tadokoro Y, et al (2001) Two mouse piwi-related genes: miwi and mili. Mech Dev 108: 121–133. 11578866 10.1016/s0925-4773(01)00499-3

[19] Deng W, Lin H (2002) miwi, a murine homolog of piwi, encodes a cytoplasmic protein essential for spermatogenesis. Dev Cell 2: 819–830. 12062093 10.1016/s1534-5807(02)00165-x

[20] Doulatov S, Notta F, Laurenti E, Dick JE (2012) Hematopoiesis: a human perspective. Cell Stem Cell 10: 120–136. 22305562 10.1016/j.stem.2012.01.006

[21] Meyer LR, Zweig AS, Hinrichs AS, Karolchik D, Kuhn RM, et al (2013) The UCSC Genome Browser database: extensions and updates 2013. Nucleic Acids Res 41: D64–69. 23155063 10.1093/nar/gks1048 PMC3531082

[22] Rosenbloom KR, Sloan CA, Malladi VS, Dreszer TR, Learned K, et al (2013) ENCODE data in the UCSC Genome Browser: year 5 update. Nucleic Acids Res 41: D56–63. 23193274 10.1093/nar/gks1172 PMC3531152

[23] Marks PA, Rifkind RA (1978) Erythroleukemic differentiation. Annu Rev Biochem 47: 419–448. 354501 10.1146/annurev.bi.47.070178.002223

[24] Ruscetti SK (1999) Deregulation of erythropoiesis by the Friend spleen focus-forming virus. Int J Biochem Cell Biol 31: 1089–1109. 10582341 10.1016/s1357-2725(99)00074-6

[25] Kanathezhath B, Mizokami M, Stanislaus S, Hounshell C, Neumayr L, et al (2011) Improved engraftment with minimal graft-versus-host disease after major histocompatibility complex-mismatched cord blood transplantation with photochemically treated donor lymphocytes. Exp Biol Med (Maywood) 236: 492–504. 21454375 10.1258/ebm.2011.010216

[26] Bracken AP, Helin K (2009) Polycomb group proteins: navigators of lineage pathways led astray in cancer. Nat Rev Cancer 9: 773–784. 19851313 10.1038/nrc2736

[27] Nolde MJ, Cheng EC, Guo S, Lin H (2013) Piwi genes are dispensable for normal hematopoiesis in mice. PLoS One 8: e71950. 24058407 10.1371/journal.pone.0071950 PMC3751959

[28] Wagner MW, Li LS, Morales JC, Galindo CL, Garner HR, et al (2008) Role of c-Abl kinase in DNA mismatch repair-dependent G2 cell cycle checkpoint arrest responses. The Journal of biological chemistry 283: 21382–21393. 18480061 10.1074/jbc.M709953200 PMC2490779

