# Supplementary material for: Deficiency of MIWI2 (Piwil4) Induces Mouse Erythroleukemia Cell Differentiation, but Has No Effect on Hematopoiesis In Vivo
Source: PLoS One. 2013 Dec 23;8(12):e82573. doi: 10.1371/journal.pone.0082573 (PMC3871168; doi:10.1371/journal.pone.0082573)
Supplement: File S1 — Supporting Documents. Figure S1. High-Performance Liquid Chromatography of Peripheral Blood from Wild Type and Homozygous MIWI2 Knockout Mice. Table S1. The Effects of Age on Blood Cell Indices. (DOCX) [file pone.0082573.s001.docx]

**Supporting Documents**

**Figure S1. High-Performance Liquid Chromatography of Peripheral Blood from Wild Type and Homozygous MIWI2 Knockout Mice.** Blood drawn from [A] wild type and [B] MIWI2 homozygous knockout mice was analyzed by high-performance liquid chromatography. Different hemoglobins were isolated by their specific retention times and quantified by the area under the curve. The percent of the total area represented by each type of hemoglobin is reported. The two images are representative samples of 4 wild type and 4 homozygous knockout mice.

Figure S1.


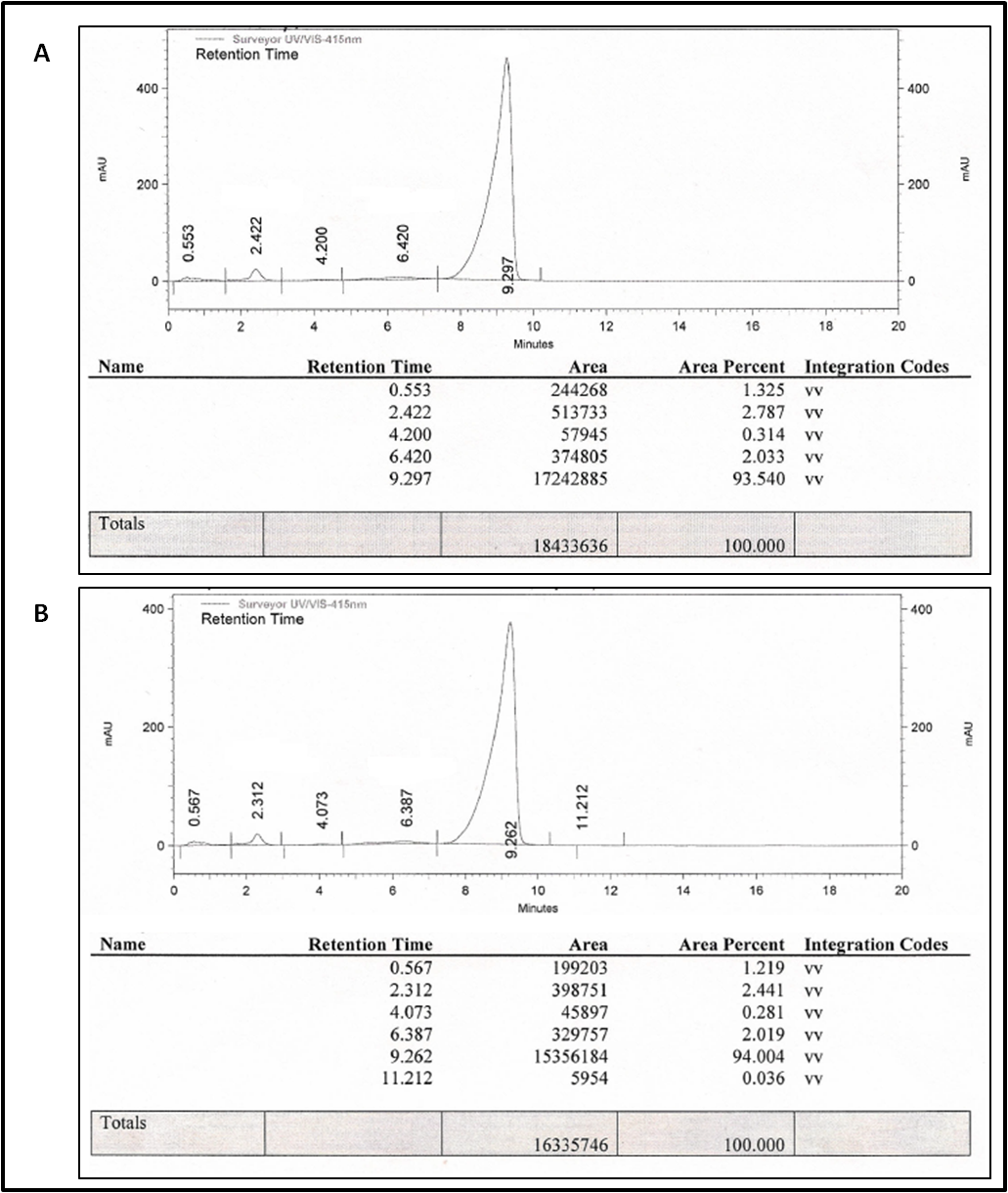


**Table S1. The Effects of Age on Blood Cell Indices**

|  | | 1 month (n=4,5) | 3 months (n=5,5) | 6 months (n=4,3) | 8 months (n=2,3) | 12 months (n=9,9) |
| --- | --- | --- | --- | --- | --- | --- |
|  |  | Hb | Hb | Hb | Hb | Hb |
| Wild Type | Average | 13.58 | 13.35 | 14.33 | 14.20 | 14.22 |
|  | Standard Dev | 1.20 | 2.85 | 0.97 | 0.92 | 0.90 |
|  |  |  |  |  |  |  |
| Homozygous MIWI2 Knockout | Average | 12.95 | 11.51 | 13.89 | 15.13 | 13.68 |
|  | Standard Dev | 2.26 | 5.48 | 0.46 | 0.97 | 1.97 |
|  | P value | 0.10 | 0.69 | 0.86 | 0.80 | 0.67 |
|  |  | WBC Count | WBC Count | WBC Count | WBC Count | WBC Count |
| Wild Type | Average | 8.9 | 5.4 | 4.7 | 4.7 | 4.8 |
|  | Standard Dev | 2.6 | 1.7 | 0.5 | 1.2 | 1.0 |
|  |  |  |  |  |  |  |
| Homozygous MIWI2 Knockout | Average | 6.2 | 5.1 | 5.1 | 9.0 | 4.8 |
|  | Standard Dev | 2.1 | 2.2 | 3.7 | 6.2 | 1.9 |
|  | P value | 0.29 | 1.0 | 0.63 | 0.80 | 0.86 |
|  |  | MCV | MCV | MCV | MCV | MCV |
| Wild Type | Average | 48.18 | 46.56 | 45.98 | 46.05 | 45.27 |
|  | Standard Dev | 0.69 | 0.69 | 0.30 | 1.34 | 0.93 |
|  |  |  |  |  |  |  |
| Homozygous MIWI2 Knockout | Average | 48.52 | 46.3 | 45.8 | 46.57 | 46.34 |
|  | Standard Dev | 1.13 | 0.70 | 0.53 | 0.46 | 1.37 |
|  | P value | 1.0 | 0.73 | 0.86 | 0.77 | 0.07 |
|  |  | Plt Count | Plt Count | Plt Count | Plt Count | Plt Count |
| Wild Type | Average | 1115.22 | 1035 | 1160.19 | 1210.99 | 1325.99 |
|  | Standard Dev | 159.18 | 157.82 | 150.40 | 24.82 | 139.09 |
|  |  |  |  |  |  |  |
| Homozygous MIWI2 Knockout | Average | 1218.02 | 1131.05 | 1365.08 | 1231.43 | 1227.19 |
|  | Standard Dev | 250.03 | 154.64 | 360.91 | 265.36 | 283.81 |
|  | P value | 0.56 | 0.55 | 0.63 | 0.80 | 0.49 |

Hb (hemoglobin concentration in g/dL). WBC Count (x1000/µL). MCV (mean corpuscular volume in fL). Plt Count (platelets x 1000/µL). The n values are given as (wild type, homozygous MIWI2 Knockout)
